# Does local soil factor drive functional leaf trait variation? A test on Neilingding Island, South China

**DOI:** 10.1186/s12862-024-02227-0

**Published:** 2024-04-10

**Authors:** Sen Tong, Juanjuan Zhang, Xueting Qiao, Buhang Li, Qiong Yang, Ping Hu, Shixiao Yu

**Affiliations:** 1 Department of Ecology, School of Life Sciences, State Key Laboratory of Biocontrol, Sun Yat-sen University, 510275 Guangzhou, China; 2 Research Institute of Sun Yat-sen University in Shenzhen, 518057 Shenzhen, China; 3 Guangdong Neilingding-Futian National Nature Reserve, 518040 Shenzhen, China

**Keywords:** Functional traits, Interspecific variation, Intraspecific variation, Leaf morphology, Stoichiometry, Subtropical forest

## Abstract

Leaf traits were affected by soil factors and displayed varietal differences in forest. However, few examples have been reported on the Island ecosystems. We comprehensively investigated 9 leaf traits (leaf length, leaf width, leaf area, SLA, leaf fresh weight, leaf C content, leaf N content, leaf K content, leaf C:N ratio) of 54 main subtropical woody species and soil parameters (soil pH, total C content, total N content, total K content, available N content, available P content, available K content and soil moisture) in Neilingding Island, Shenzhen, southern China. Intra-and interspecific variation of leaf traits were measured and their correlations with soil parameters were explored. The interspecific variations of leaf C:N ratio, leaf N content and leaf fresh weight were higher than their intraspecific variations. The intraspecific variation of leaf K content was larger than that of interspecific one, accounting for 80.69% of the total variance. Positive correlations were found among intraspecific coefficients of variations in leaf morphological traits. The correlation analysis between the variation of intraspecific traits and the variation of soil parameters showed that changes in soil factors affected leaf morphology and stoichiometry. The interaction between soil moisture and soil available P content was the key factor on intraspecific variations of leaf traits including leaf area, leaf fresh weight, leaf C and leaf K content. We concluded that leaf traits of plants in the island were tightly related to soil parameters. Soil parameters, especially soil moisture and available P content, affected plant leaf morphology and stoichiometry at the local scale.

## Introduction

Changes in soil properties can affect leaf traits [1, 2]. With changes in soil fertility, responses in leaf traits tend to vary among individuals within species and among species. Understanding these responses can help us to understand adaptation strategies of plants and the underlying mechanisms. Nitrogen (N) and phosphorus (P) are the most common nutrients limiting net primary productivity in terrestrial ecosystems. Due to the sedimentation of nitrogen, the soil nitrogen content is relatively high; while the soil phosphorus mainly comes from rock weathering and the content is low. Plant productivity in tropical forests is generally regarded as P limited, rather than N limited, because soil P availability generally declines with bedrock weathering and soil age [3] and quaternary glaciation exposed fresh bedrock over a large area of temperate and boreal regions, but not in the tropics [4]. However, tropical forests maintain the greatest plant biomass and the fastest rates of many biological processes (i.e., decomposition, N transformation) on Earth. Therefore, identifying the strategies that tropical plants have evolved to use P efficiently under low soil P availability is an important topic in plant ecology.Leaf trait variation in response to environmental changes can reflect adaptive strategy of plants.

Neilingding Island is part of Neilingding-Futian National Nature Reserve in Guangdong province, south China. Its climax vegetation type is subtropical evergreen broad-leaved forest. The island’s fragile ecosystem and the low soil organic matter content brought huge challenges to plant growth and community stability. However, few studies on plant adaptive strategy for environmental stress have been conducted on this Island. By exploring the relationships between leaf traits and soil factors, this research aims to reveal the adaptation mechanism of plants in Neilingding Island and setup a scientific basis for plant resources management.

## Materials and methods

### Study sites

**Fig. 1:**
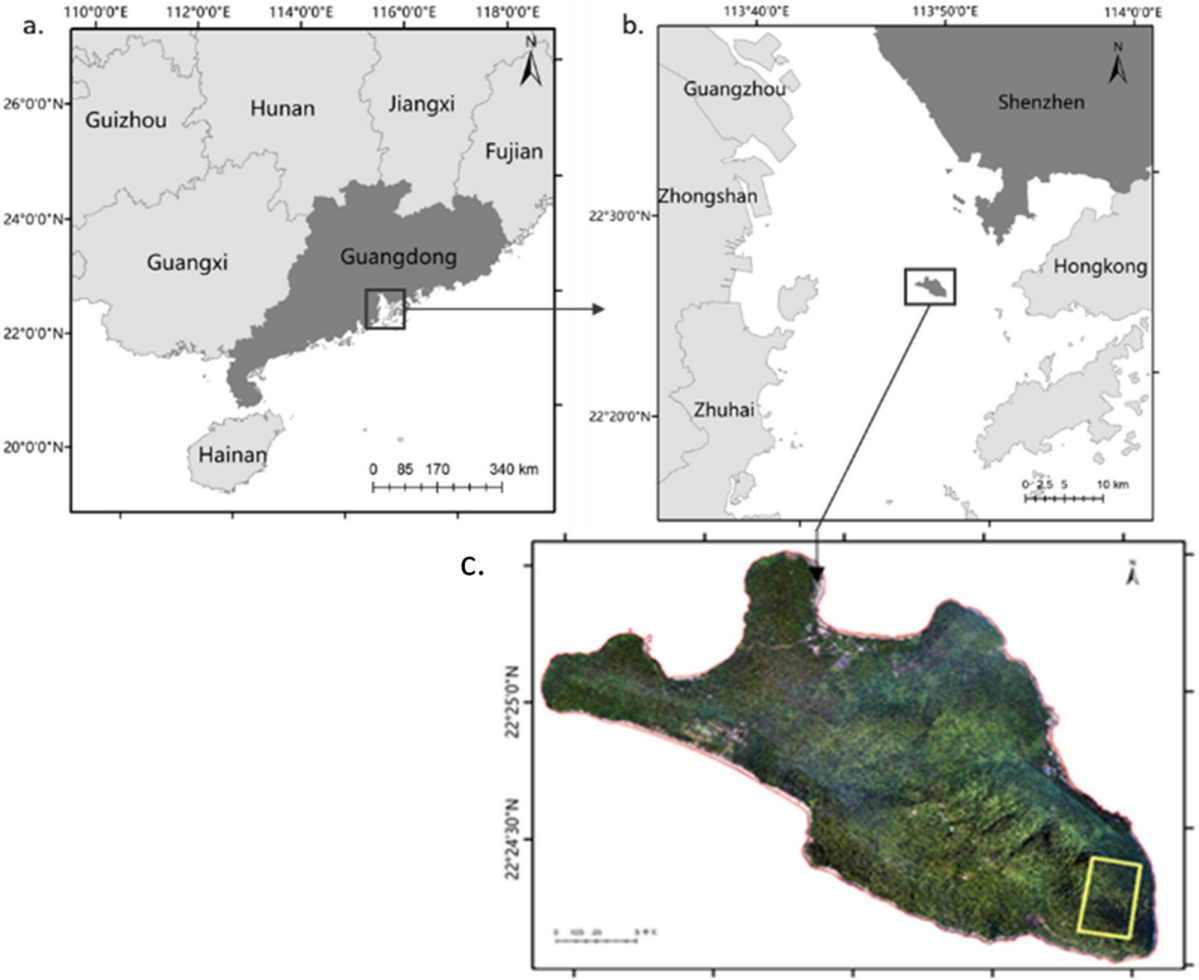
Location of the 15-ha plot in Neilingding (Rectangle is sampling plot). **a**, **b** were followed the map of China (https://bzdt.ch.mnr.gov.cn), c was drew based on our unmanned aerial vehicles photogrammetry in 2020

Neilingding Island is located in the southwest of Shekou Peninsula (113° 46 ‘18 “∼ 113° 49’ 49"E, 22° 23 ‘49 “∼ 22° 25’ 3"N). It is the largest island in Shenzhen, Guangdong Province, with a total area of 4.98 km^2^ (Fig. 1). The landform of Neilingding Island is hilly, which is generally high in the middle and low around. The climate type of Neilingding Island is subtropical monsoon climate. The island is hot and humid all the year round, with an average annual temperature of 22.0℃ to 22.4℃, about 2000 mm average annual rainfall. The island has obvious dry season and wet season, with precipitation mainly concentrated from April to September. The annual total sunshine is about 2,000 h [5]. The soil is composed of metamorphic granite and sandstone, and the type of soil is mainly coastal sandy soil, cultivated and red soil (pH is about 4.0 to 6.0). The content of soil organic matter is low, and the capacity of fertilizer-preserving and fertilizer-supplying is weak.

### Leaf trait measurements

From late 2019 to early 2020, we constructed a 15-hectare plot on Neilingding Island. The investigator identify the plant with the help of databases such as FLORA oF China. Species were identified by Dr. Buhang Li and some species were identified by Dr. Qiang Fan from Herbarium of Sun Yat-sen University (SYSU) with plant specimens collected in the field. Specimens were deposited in Herbarium of Sun Yat-sen University (SYSU). There were totally 78 plant species (Table 1), with 54 woody plant species from 35 families were recorded. Plant individuals with DBH≥1 cm were censused, with height, DBH, and the location in the plot. For each tree species, target individuals with different breast diameters were selected for sampling of leaves. The number of samples of the species with large populations is greater than or equal to 10, while samples of the species with small populations were obtained as much as possible within the available range to ensure that the number of individuals is 5～10. The location of these sampling trees were recorded. The total number of sampled individuals is 479. As shown in the figure, the individual target tree species are randomly distributed in the plot.

**Table 1:** List of 78 species

Species	Family	Species	Family
*Acacia confusa*	Fabaceae	*Glochidion puberum*	Euphorbiaceae
*Ailanthus fordii*	Simaroubaceae	*Glycosmis parviflora*	Rutaceae
*Alangium kurzii*	Alangiaceae	*Ilex kwangtungensis*	Aquifoliaceae
*Antidesma bunius*	Euphorbiaceae	*Ilex rotunda*	Aquifoliaceae
*Antirhea chinensis*	Rubiaceae	*Itea chinensis*	Saxifragaceae
*Aporosa dioica*	Euphorbiaceae	*Koelreuteria bipinnata*	Sapindaceae
*Aralia decaisneana*	Araliaceae	*Laurocerasus zippeliana*	Rosaceae
*Archidendron lucidum*	Leguminosae	*Litchi chinensis*	Sapindaceae
*Ardisia crenata*	Myrsinaceae	*Litsea monopetala*	Lauraceae
*Artocarpus hypargyreus*	Moraceae	*Litsea rotundifolia*	Lauraceae
*Atalantia buxifolia*	Rutaceae	*Litsea verticillata*	Lauraceae
*Bischofia javanica*	Euphorbiaceae	*Macaranga tanarius*	Euphorbiaceae
*Breynia fruticosa*	Euphorbiaceae	*Mallotus paniculatus*	Euphorbiaceae
*Bridelia tomentosa*	Euphorbiaceae	*Mallotus philippensis*	Euphorbiaceae
*Brucea javanica*	Simaroubaceae	*Melia azedarach*	Meliaceae
*Callicarpa nudiflora*	Verbenaceae	Microcos paniculata	Tiliaceae
*Casearia glomerata*	Salicaceae	*Oroxylum indicum*	Bignoniaceae
*Celtis sinensis*	Ulmaceae	*Phoenix loureiroi*	Arecaceae
*Chukrasia tabularis*	Meliaceae	*Psidium guajava*	Myrtaceae
*Cinnamomum camphora*	Lauraceae	Psychotria rubra	Rubiaceae
*Citrus limon*	Rutaceae	*Pterospermum heterophyllum*	Malvaceae
*Citrus reticulata*	Rutaceae	*Randia wallichii*	Rubiaceae
*Claoxylon indicum*	Euphorbiaceae	*Sageretia thea*	Rhamnaceae
*Clerodendrum cyrtophyllum*	Verbenaceae	*Sapium sebiferum*	Euphorbiaceae
*Cratoxylum cochinchinense*	Hypericaceae	*Schefflera octophylla*	Araliaceae
*Desmos chinensis*	Annonaceae	*Scolopia chinensis*	Salicaceae
*Dimocarpus longan*	Sapindaceae	*Sterculia lanceolata*	Sterculiaceae
*Diospyros vaccinioides*	Ebenaceae	*Strophanthus divaricatus*	Apocynaceae
*Emmenopterys henryi*	Rubiaceae	*Strychnos angustiflora*	Loganiaceae
*Euonymus alatus*	Celastraceae	*Syzygium levinei*	Myrtaceae
*Euonymus laxiflorus*	Celastraceae	*Tarenna attenuata*	Rubiaceae
*Ficus fistulosa*	Moraceae	*Tarenna mollissima*	Rubiaceae
*Ficus hirta*	Moraceae	*Thevetia peruviana*	Apocynaceae
*Ficus hispida*	Moraceae	*Trema tomentosa*	Ulmaceae
*Ficus microcarpa*	Moraceae	*Trigonostemon wui*	Euphorbiaceae
*Ficus variegata*	Moraceae	*Ventilago leiocarpa*	Rhamnaceae
*Fissistigma uonicum*	Annonaceae	*Viburnum odoratissimum*	Caprifoliaceae
*Flueggea virosa*	Euphorbiaceae	*Vitex quinata*	Verbenaceae
*Glochidion macrophyllum*	Euphorbiaceae	*Zanthoxylum avicennae*	Rutaceae

We measured 9 plant leaf functional traits [6], inlcuding leaf length (LL, cm), leaf width (LW, cm), leaf area (LA, cm^2^), specific leaf area (SLA, cm2/g), leaf fresh mass (LFM, g), leaf nitrogen content (LN, g/kg), leaf carbon content (LC, g/kg), leaf potassium content (LK, g/kg), leaf carbon to nitrogen ratio (Leaf C:N). Leaf area was calculated by digitally scanning each leaf individually and then analyzing the images using an ImageJ program. Dry weight was measured after a minimum of 48 h in a drying oven at 65 °C. SLA was calculated as the ratio of leaf area to dry leaf mass.

### Soil sampling and determination

We selected 625 sites (marked geographic sites) from the 15-hectare plot, and got soil samples with a sampling depth from 0$\sim$20 cm after removing the plants and litters in the topsoil. Use soil temperature and humidity instrument to measure soil moisture (soil M, %). The soil samples were sent to South China Botanical Garden Chinese Academy of Sciences, for analyzing, including soil pH, total organic carbon content (TC, g/kg), total nitrogen content (TN, g/kg), total phosphorus content (TP, g/kg), effective nitrogen content (AN, g/kg), available phosphorus content (AP, g/kg), effective potassium content (AK, g/kg), soil moisture (%). The average of soil pH is 4.6, which is basically consistent with the soil pH (pH < 5) of the mainland subtropical forest in south China.

### Statistical analyses

#### Interspecific variation and intraspecific variation of traits

In order to obtain the proportion of intraspecic variation and interspecific variation in the total variation of each leaf trait, we fitted a linear mixed-effects models for each leaf trait through R package “lme4” [7]. The nested model included both intraspecic and interspecific random effects of leaf traits, and the significance was set as *P* < 0.05. Leaf trait variation was decomposed into interspecific and intraspecific variation. The formula is as follows:

$${{\rm{y}}_{{\rm{ij}}}}\,{\rm{ = }}\,{{\rm{\mu }}_{\rm{i}}}\,{\rm{ + }}\,{{\rm{\varepsilon }}_{{\rm{ij}}}}$$

Where y_ij_is the leaf trait value of the j’th individual from i’th species,, ${\mu }_{i}$ is a random variance to explain the variation of a species leaf traits, ${\epsilon }_{ij}$ is the residual.

The intraspecific and interspecific variation of each trait were calculated by the coefficient of variation. The intraspecific variation was calculated by the measured trait value of all sampling individual of a species, while interspecific variation was calculated by the average trait value of each species. The formula is as follows:

$$\eqalign{& {\rm{Coefficient}}\,{\rm{of}}\,{\rm{Variation}}\,{\rm{for}}\,{\rm{leaf}}\,{\rm{traits}}\,{\rm{(CV)}} \cr & = \,{\rm{Standard}}\,{\rm{Deviation}}\,{\rm{of}}\,{\rm{traits}}\,{\rm{(SD)}} \cr & {\rm{/Mean}}\,{\rm{value}}\,{\rm{of}}\,{\rm{traits}}\,{\rm{(M)}} \cr} $$

#### Regression analysis

Multiple linear regression model was used to analyse the data. Individual leaf traits was regared as response, 8 soil factors as candidate variables for interpretation, and the focal tree species as random effects:

$${y_{ij}}\, = \,{\beta _0}\, + \,{\beta _1}{e_{ij}}\, + \, \ldots {\beta _7}{e_{7ij}}\, + \,{\beta _8}e_{8ij}^{}\, + \,{\mu _i}\, + \,{\varepsilon _{ij}}$$

Where ${\text{y}}_{ij}$ is the leaf trait value of the j’th individual from i’th species, ${\beta }_{1}$,${\beta }_{2}$,…,${\beta }_{8}$ correspond to the fixed effects of 8 soil variables respectively. ${\beta }_{0}$ is a fixed intercept, and ${\mu }_{i}$ is a random intercept.

A simple linear model was used to fit the interspecific relationship between leaf traits and soil factors:

$${\mu _i} = {\alpha _0} + {\alpha _1}{E_{1i}} + \cdots + {\alpha _8}{E_{8i}} + {\varepsilon _i}$$

$$ {E}_{1i}$$,$$ {E}_{2i}$$,…,$$ {E}_{8i}$$ is the average value of each soil variables in all sampling plots for i’th species. $$ {\alpha }_{1},{\alpha }_{2}$$,…,$$ {\alpha }_{8}$$correspond to the fixed effects of 8 soil variables respectively. The effects of different environmental predictors are quantified as corresponding local slopes.

Relation between leaf traits and soil factors might not exhibit a simple linear relation. We used Akaike’s Information Standard (AIC) through the R package “lmetest” to determine the most relevant generalized linear model for each pair of coefficient of variation (CV) with a leaf trait and a soil variable. The interaction of soil factor changes on leaf trait variation was analyzed based on “pred” of R package “sjmisc”.

## Results

### Interspecific and intraspecific trait variation

**Fig. 2:**
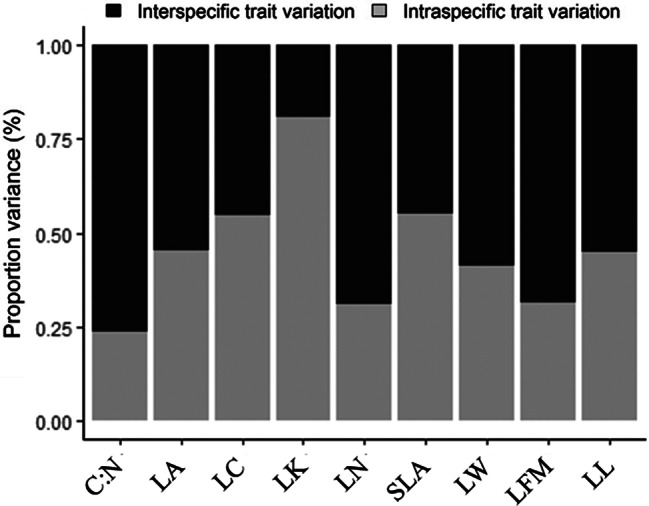
The proportion of variance attributed to inter-(dark grey) and intraspecific variation (light gray) of the 9 leaf traits. C:N, the ratio of leaf carbon to nitrogen; LA, leaf area; LC, leaf carbon content; LK leaf potassium content; LN, leaf nitrogen content; SLA, specific leaf area; LW, leaf width; LFM, leaf fresh mass; LL, leaf length

For leaf C:N, LN and LFW, interspecific variation was stronger than intraspecific variation, with the former accounting for more than 65% of the total variance. For LK, however, intraspecific variation was stronger, accounting for more than 80% of the total variance. For the other 5 traits, inter-and intraspecific variation were similarly strong (Fig. 2).

### Correlation between leaf traits

**Fig. 3:**
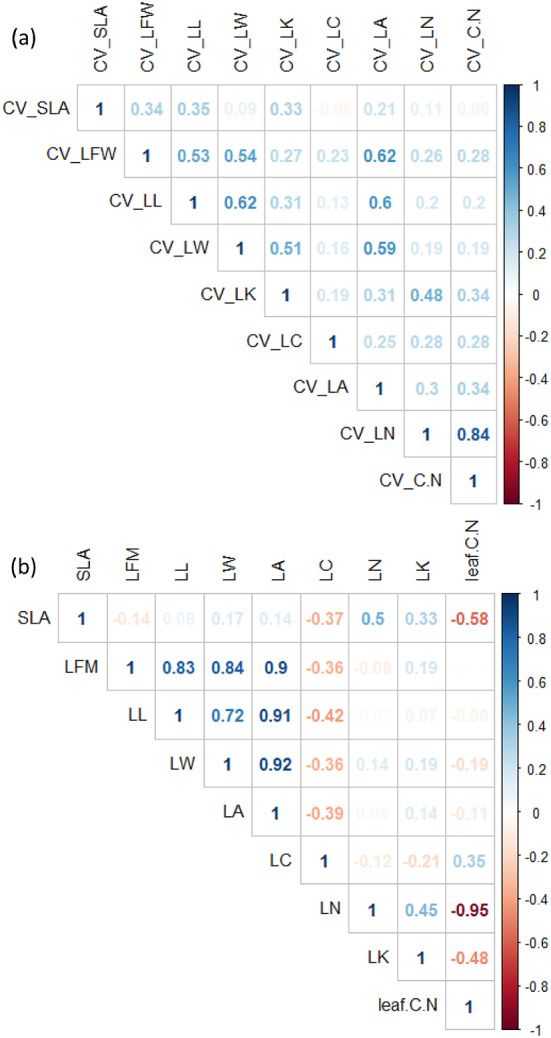
Pairwise Spearman rank correlation coefficients for nine leaf traits measured (**a**) intraspecific trait variation, (**b**) species-mean trait values

SLA is positively correlated with LN and LK, and negatively correlated with LFM, LC, and C/N (Fig. 3). The morphological traits, including LA, LL, LW and LFM, and significantly positively correlated with each other. For the chemical traits, LN and LK were positively correlated, and they were negatively correlated with leaf C:N. LC was not significantly correlated with other chemical traits (Fig. 3).

Spearman’s pairwise rank correlation coefficient test (Fig. 3) for the intraspecific coefficient of variation (CV trait) of leaf traits shows that the intraspecific coefficient of variation of each trait is positively correlated. The results showed that the stronger the correlation between leaf traits, the stronger the correlation between leaf traits within-species variation. Leaf morphological traits often show a strong positive correlation between intraspecific variation, which may be due to morphological traits. Variations in leaf are often strongly linked. Variations in one trait often lead to variations in other morphological traits. SLA did not significantly correlated with intraspecific variation in other leaf traits. Changes in LC have a greater impact on the ratio of leaf carbon to nitrogen. The relative coefficient of the paired Spearman relative coefficient of LN intraspecific variation and leaf C/N of intraspecific variation exceeds 0.64. The correlation between leaf stoichiometric traits and morphological traits coefficient of variation is not significant.

### Interaction effects of soil factors

**Fig. 4:**
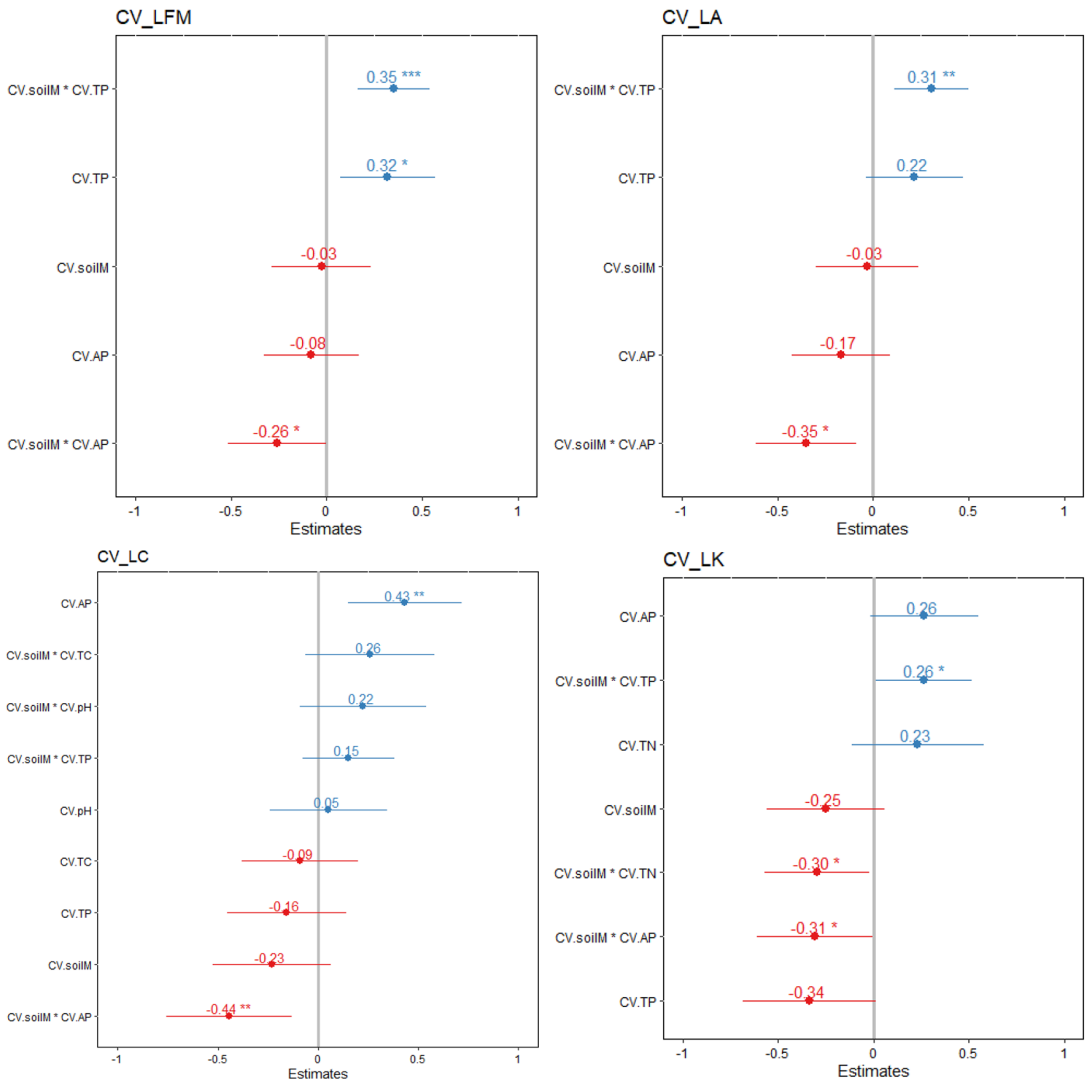
Estimated standardized effect sizes of the interaction between leaf traits and soil factors. Circles and lines show the means and 95% credible intervals of the coefficients. If the 95% credible intervals excluded zero respectively, circles indicate statistically significant effects) (*p*<0.01 **, *p*<0.05 *)

The CV association with soil moisture and available phosphorus content was significant for LA, LFW, and LK (Fig. 4; *p* < 0.05). LN, leaf C/N ratio intraspecific variation did not significantly correlated with variation in soil factors (*p* > 0.05), not also for relationship between intraspecific variation of SLA and soil factors. CV of LC significantly correlated with the interaction of CVs of soil available phosphorus and soil moisture (*p* < 0.01).

The selected traits did not significantly correlated with soil pH changes (*p* > 0.05). There is also no significant correlation between the selected leaf traits and soil nitrogen content (*p* > 0.05). The total soil nitrogen content was relatively high, and the overall variation was small. It did not significantly affect the plant leaf traits.

**Fig. 5:**
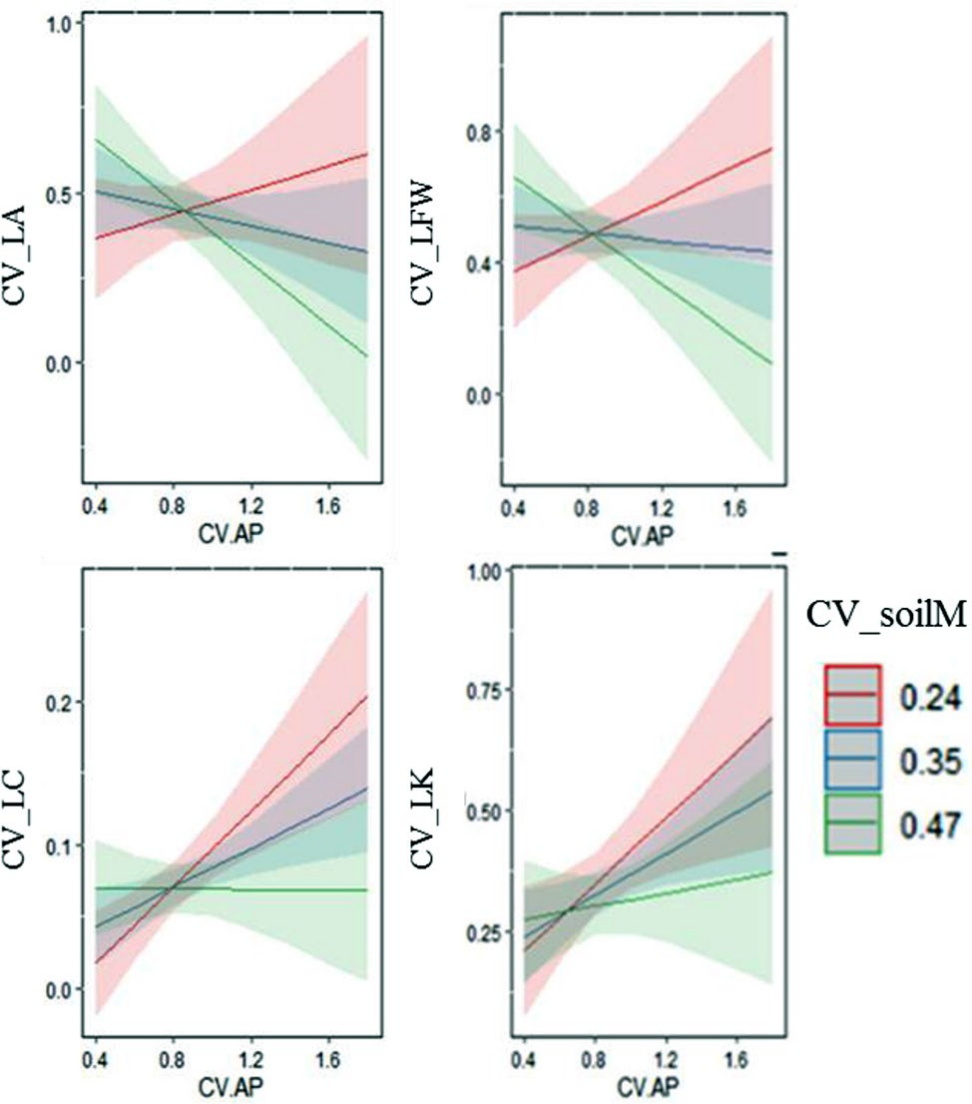
Effects of interaction between soil available phosphorus and soil moisture variation on prediction of intraspecific variation: (**a**) LA, (**b**) LFW, (**c**) LC, (**d**) LK

At lower soil moisture variation (CV = 0.24), the greater the change in soil available phosphorus content is, the greater variation in the leaf area, leaf fresh weight, LC and LK within species (Fig. 5). At a moderate level of soil moisture variation (CV = 0.35), the variation of leaf area and leaf fresh weight did not change greater with the increasing variation of soil available phosphorus, while the intraspecific variation of LC and LK increased. At a high level of soil moisture variation (CV = 0.47), the intraspecific variation of leaf traits was not sensitive to the changes in soil available P (Fig. 5).

## Discussion

Qualitative regulation of abiotic stress within species is common in plants, and it is evident in many plant traits. While soil total phosphorus content is low, and the stress of available phosphorus may change the expression of intraspecific traits of woody plants on Neilingding Island to a certain extent. This is because the acquisition and utilization of phosphorus is very important for plant growth [8–10], changes in the environmental supply of phosphorus will affect the expression of highly conserved genes [11], and use differences in plant functional traits to affect plant phosphorus Access [12–14]. There was a significant correlation between the intraspecific variation of leaf traits and soil available phosphorus, confirming that woody plants adapt to changes in soil available phosphorus content through individual trait variation. The interaction of species related to phosphorus acquisition is to maintain the ecosystem stable. The key mechanism of diversity provides evidence for how plants in tropical and subtropical regions adapt to low-phosphorus soil environments.

The soil P is low on Neilingding Island. Although we provide more reliable evidence that the unique influence of soil P is the main driving factor for the leaf traits variation. It is still a major challenge to understand the physiological, ecological and evolutionary mechanisms of the relationship between plant traits and the environment. As the kwy limiting factor of plants on Neilingding Island, soil P plays an important role in maintaining the stability and diversity of community.

## Conclusion

We find that soil moisture and available P on Neilingding Island had great influences on intraspecific variation of leaf traits. Plants adapt to changes in soil available P by intraspecific variation. Soil factors, especially soil moisture and soil available phosphorus, affected nutrient concentration and morphology of leaves, and drive intraspecific variation of leaf morphology and stoichiometry. We have improved the ecosystem understanding and has an important reference role for the plant resources protection and subsequent research on Neilingding Island. P, as a key limiting factor for plants on Neilingding Island, leads leaf morphology and stoichiometry to vary within a certain range, thereby increasing the diversity of intra-and inter-species trait combinations, and improving the adaptability of different species in the community.

## Data Availability

The datasets generated during and/or analysed during the current study are available from the corresponding author on reasonable request.
